# The properties of MOF-Zn_2_(EBNB)_2_(BPY)_2_·2H_2_O and its basic study of loading methadone

**DOI:** 10.1186/s13065-020-00709-y

**Published:** 2020-09-18

**Authors:** Deng Linxin, Li Song, Sun Xuehua

**Affiliations:** 1 Department of Drug Control, Key Laboratory of Narcotics Assay and Control Technology Ministry of Public Security, Yunnan Police College, Kunming, 650223 China; 2 College of Science, Yunnan Agricultural University, Kunming, 650201 China

**Keywords:** MOFs, Methadone, Drug loading, Drug release, Cytotoxicity

## Abstract

The ligands of (E)-bis(p-3-nitrobenzoic acid) vinyl (C_16_H_10_N_2_O_8_) were synthesized in three steps, and then the MOF-Zn_2_(EBNB)_2_(BPY)_2_·2H_2_O was synthesized by solvothermal method. This structure was characterized by X-ray single crystal diffraction, SEM and TG. The drug loading and in vitro release of Zn_2_(EBNB)_2_(BPY)_2_·2H_2_O were also studied with Methadone as model drug. The results show that the highest loading amount of Zn_2_(EBNB)_2_(BPY)_2_·2H_2_O to Methadone was 0.256 g/g, and the drug delivery system was a two-phase mode. The results of in vitro cytotoxicity test show that Zn_2_(EBNB)_2_(BPY)_2_·2H_2_O has good biocompatibility.

## Introduction

Methadone is the main drug in current opioid drugs addiction replacement therapy [1]. Methadone can bind to opioid receptors competitively in human body, and can produce cross dependence and tolerance with opioids such as heroin, thus it can reduce the sensitivity of opioid receptors in drug dependent patients [2]. At the same time, combined with psychotherapy, behavioral intervention and other comprehensive measures, methadone can eventually reduce the harm of drugs and the demand for drugs to addicts [3].

The dosage of methadone has been a controversial issue. There are many studies supporting the use of high-dose methadone, that is to say, the higher the dosage of methadone, the longer the holding time of patients, and the better effect of alternative maintenance therapy [4]. However, methadone itself is addictive, and long-term or high-dose use is easy to lead to addiction [5]. Nevertheless, the action time of low-dose methadone (such as 2.5–5 mg) is only 6-8 h [6], which is not convenient for clinical use.

At present, the research hotspots of methadone replacement maintenance therapy focus mainly on the cognitive survey of patients with methadone maintenance treatment [7], the operation of methadone outpatient service [8], the retention rate and its influencing factors in methadone maintenance treatment [9], and the cost-effectiveness of methadone maintenance treatment [10].

As one of the advanced frontier materials, metal organic frameworks(MOFs) is the fastest developing direction of coordination chemistry in recent 10 years [11]. It has a super molecular network structure [12], and has the advantages of both inorganic and organic compounds [13]. The structure of this kind of frame material can realize diversified design and adjustable pore size or structure. So it can realize semi directional design synthesis [14]. The research of this kind of molecular functional materials has spanned many fields such as crystallography, materials science, inorganic chemistry, coordination chemistry, organic chemistry, etc. [15]. As a new type of nano porous material, comparing with activated carbon and zeolite, MOFs have been widely used in the fields of gas capture, storage and separation [16]; drug release [17]; optical, magnetic, electrical science [18]; catalytic science [19]; chiral resolution [20] and so on.

Zn_2_(EBNB)_2_(BPY)_2_·2H_2_O is a new type of 3D porous MOFs material [21]. It has excellent gas adsorption performance in study of many kinds of gas molecules, but it has not been reported as a drug carrier. In this paper, we use methadone as the model drug, and optimized the preparation process of the drug carrier by two factors. We investigated the in vitro release characteristics and in vitro cytotoxicity experiment of the drug carrier Zn_2_(EBNB)_2_(BPY)_2_·2H_2_O, which provided the foundation for its development and application as a drug carrier.

## Results and discussion

### Chemistry

**Scheme 1:**
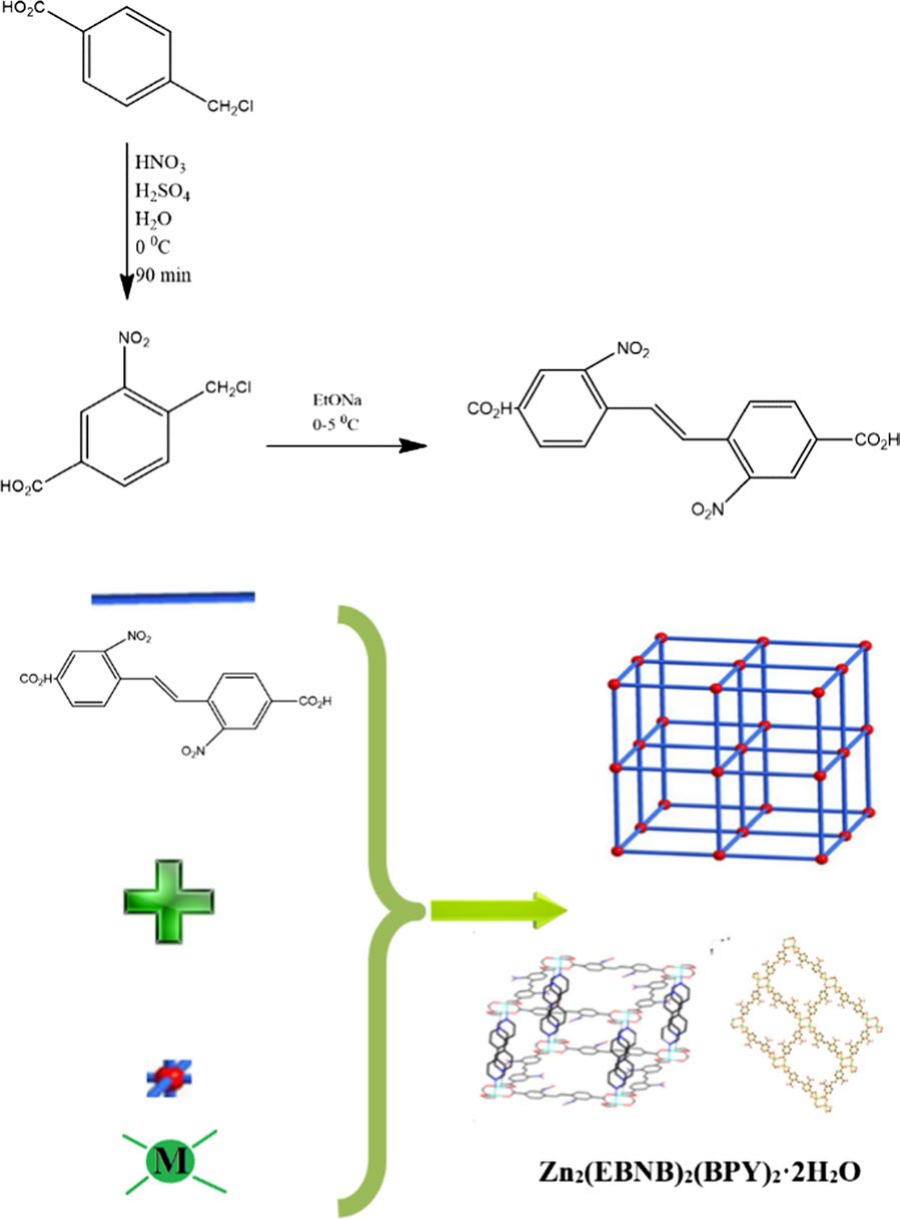
synthetic route of (E)-di(p-3-nitrobenzoic acid) ethylene (C_16_H_10_N_2_O_8_) and Zn_2_(EBNB)_2_(BPY)_2_·2H_2_O

The synthesis of Zn_2_(EBNB)_2_(BPY)_2_·2H_2_O by solvothermal method began with the synthesis of (E)—bis (p-3-nitrobenzoic acid) ethylene ligand (C_16_H_10_N_2_O_8_) which was synthesized by Nitrification and coupling from p-chloromethylbenzoic acid (Scheme 1).

### Crystal structures of Zn_2_(EBNB)_2_(BPY)_2_·2H_2_O

**Fig. 1:**
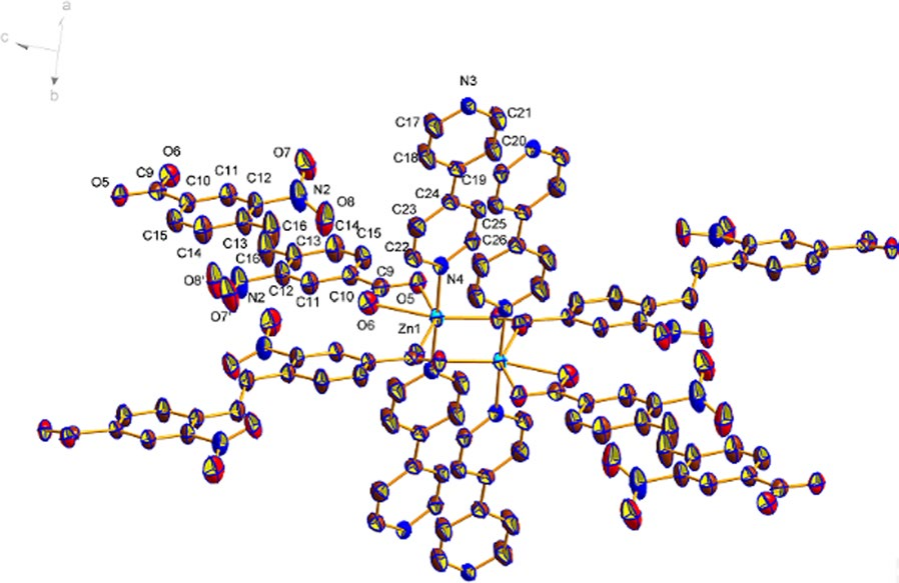
Secondary building unit of Zn_2_(EBNB)_2_(BPY)_2_·2H_2_O

**Fig. 2:**
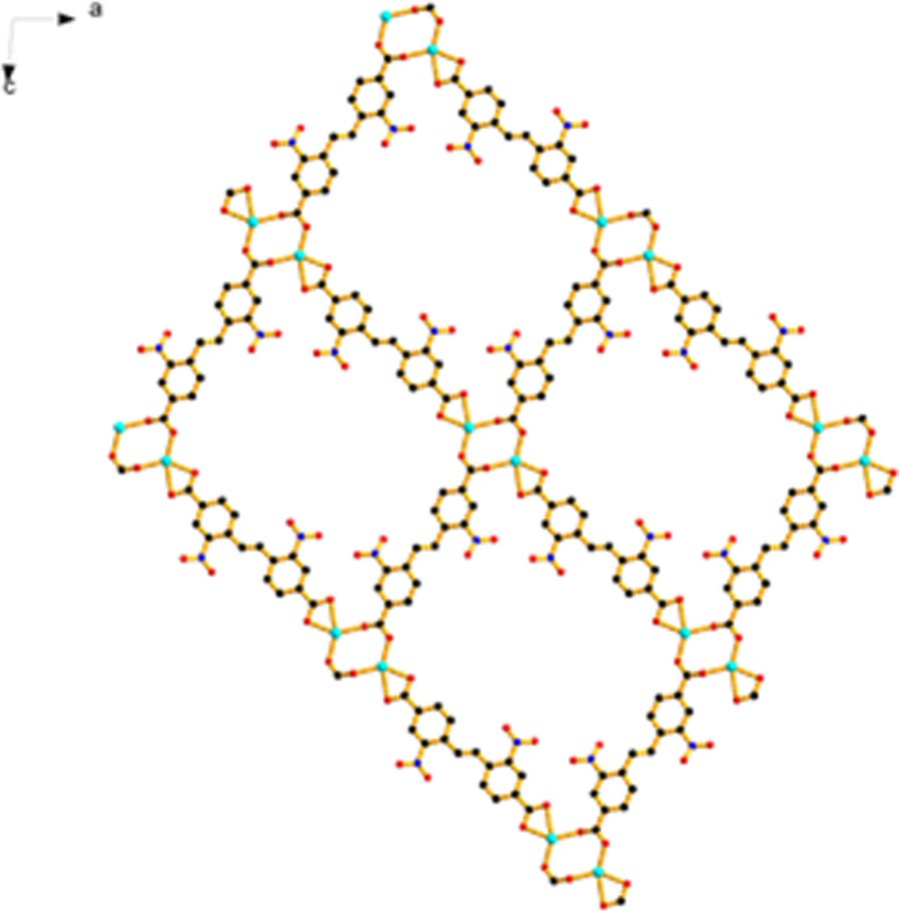
2D layer structure of Zn_2_(EBNB)_2_(BPY)_2_·2H_2_O

**Fig. 3:**
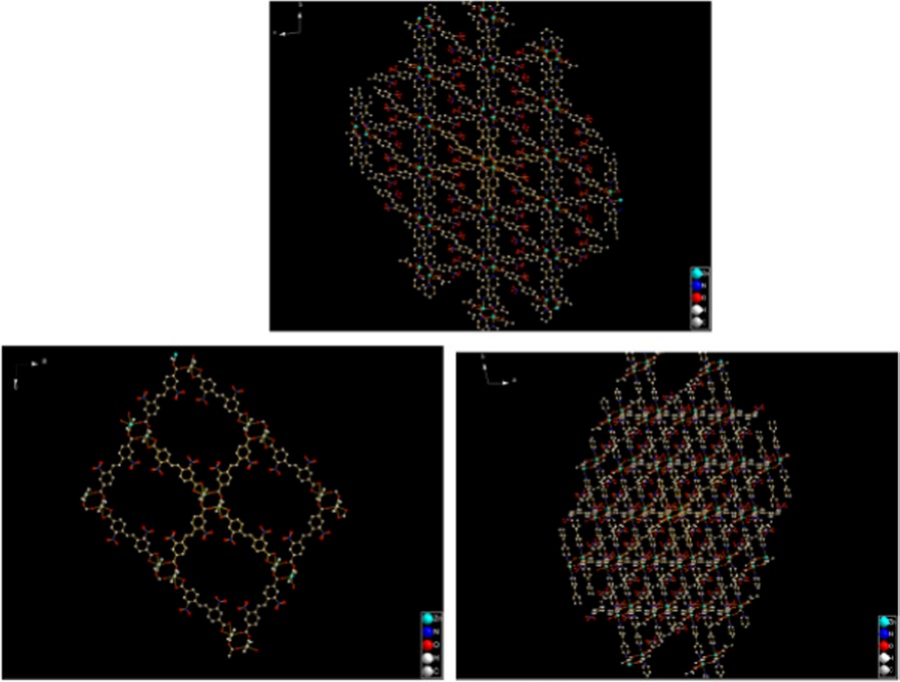
Three dimensional structure of Zn_2_(EBNB)_2_(BPY)_2_·2H_2_O

Single X-ray crystal diffraction analysis reveals that compound 1 crystallizes in the P-1 space group, and possesses an extended 3D framework with a novel dinuclear Zinc clusters as secondly build unit. In dinuclear Zinc unit, each Zn^2+^ is connected by four oxygen atoms, two of which come from the same EBNB ligand, as shown in Fig. 1. The distance of Zn–O is 2.03 Å. The remaining two oxygen atoms come from two different EBNB ligand respectively (Fig. 1), and the distance of Zn–O is 2.13 and 2.17 Å respectively. The EBNB also have two coordination modes with the Zn^2+^, first one is the carboxylic oxygen atom links to one Zn^2+^, and the other is two oxygen atoms connect to two Zn^2+^ respectively. Each cluster unit is linked with neighboring units through four EBNB ligands to form a 2D layer (Fig. 2). Four BPY molecules serve as pillar ligands to coordinate with the outer Zn atoms which gives raise to 3D framework (Fig. 3).

**Fig. 4:**
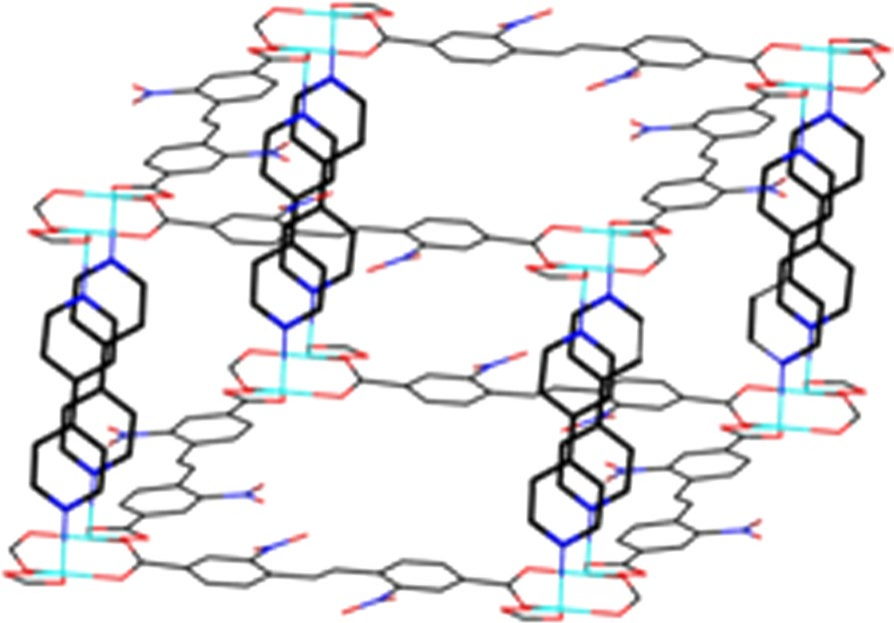
The frame structure of Zn_2_(EBNB)_2_(BPY)_2_·2H_2_O

Figure 1 also shows that a single (E)-di(p-3-nitrobenzoic acid) ethylene ligand is coordinated with two Zn (II) ions along the AC plane, and each Zn (II) ion is also coordinated with an oxygen coordination atom in a water molecule and two (E)-di(p-3-nitrobenzoic acid) ethylene ligands, thus extending outward to form a 2D plane. Along the c-axis direction, the layers and the interlayer are connected with Zn (II) ion via bipyridine column ligand to build a 3D network structure. And finally, a 3D metal organic framework material with one-dimensional pore structure is formed (Figs. 2, 3 and 4).

### Microscope and SEM image of Zn_2_(EBNB)_2_(BPY)_2_·2H_2_O

**Fig. 5:**
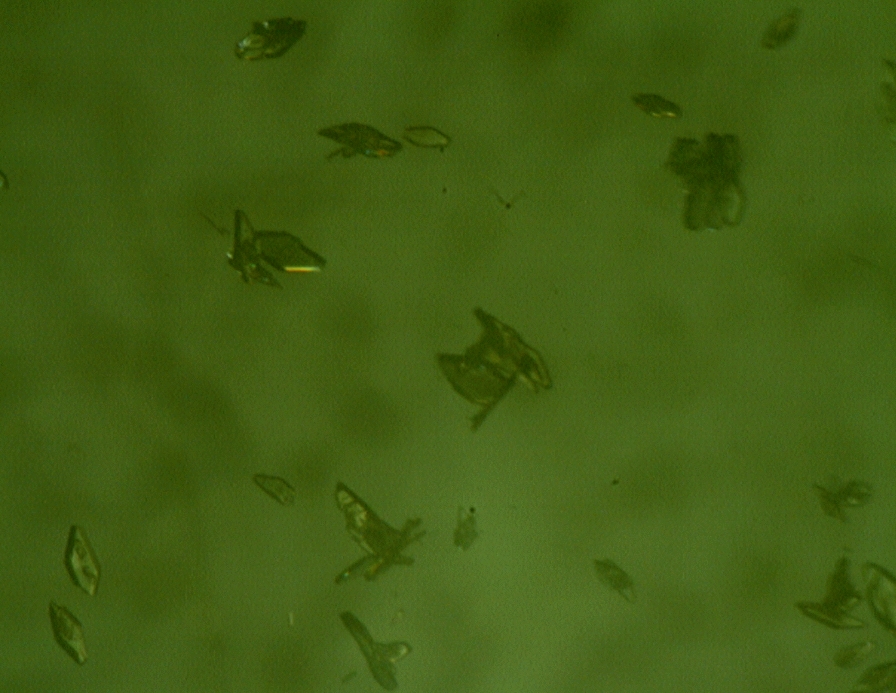
Microscopic image of Zn_2_(EBNB)_2_(BPY)_2_·2H_2_O (using Oubo SK2009)

**Fig. 6:**
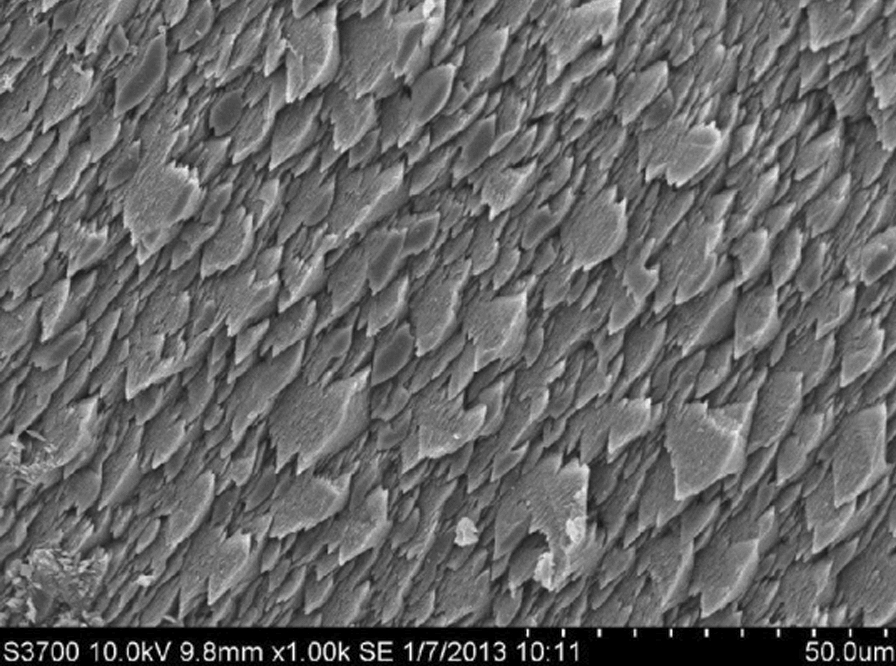
SEM image of Zn_2_(EBNB)_2_(BPY)_2_·2H_2_O (using Hitachi S-3700 N)

The morphology of Zn_2_(EBNB)_2_(BPY)_2_·2H_2_O was characterized by microscope and SEM. Figure 5 is a microscope picture of Zn_2_(EBNB)_2_(BPY)_2_·2H_2_O. It can be seen from Fig. 5 that it is yellow diamond crystal. Figure 6 shows the surface morphology of Zn_2_(EBNB)_2_(BPY)_2_·2H_2_O in SEM. It can be seen from Fig. 6 that the surface structure of Zn_2_(EBNB)_2_(BPY)_2_·2H_2_O is fish scale.

### Powder XRD characterization of Zn_2_(EBNB)_2_(BPY)_2_·2H_2_O

**Fig. 7:**
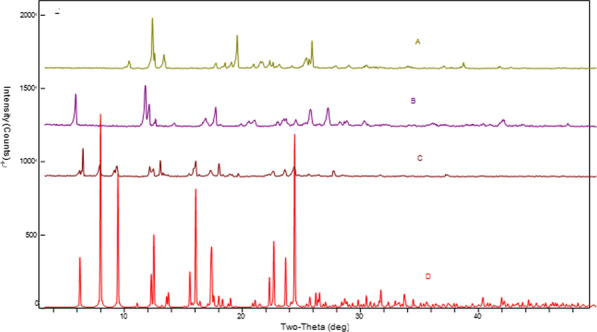
Powder XRD characterization of Zn_2_(EBNB)_2_(BPY)_2_·2H_2_O

Figure 7 is the powder XRD characterization of Zn_2_(EBNB)_2_(BPY)_2_·2H_2_O which are actual measured, software simulation’s and ligand’s. Among it, A is the ligand bipyridine’s XRD, B is the (E)-di(p-3-nitrobenzoic acid) ethylene ligand’s XRD, C is the Zn_2_(EBNB)_2_(BPY)_2_·2H_2_O’s actual measured XRD, and D is the Zn_2_(EBNB)_2_(BPY)_2_·2H_2_O’s simulated XRD. It can be seen from the figure that the measured value of Zn_2_(EBNB)_2_(BPY)_2_·2H_2_O is in good agreement with the simulated value of the software, which shows that the compound is a pure phase, and there are obvious differences compare with the two ligands.

### Thermal analysis of Zn_2_(EBNB)_2_(BPY)_2_·2H_2_O

**Fig. 8:**
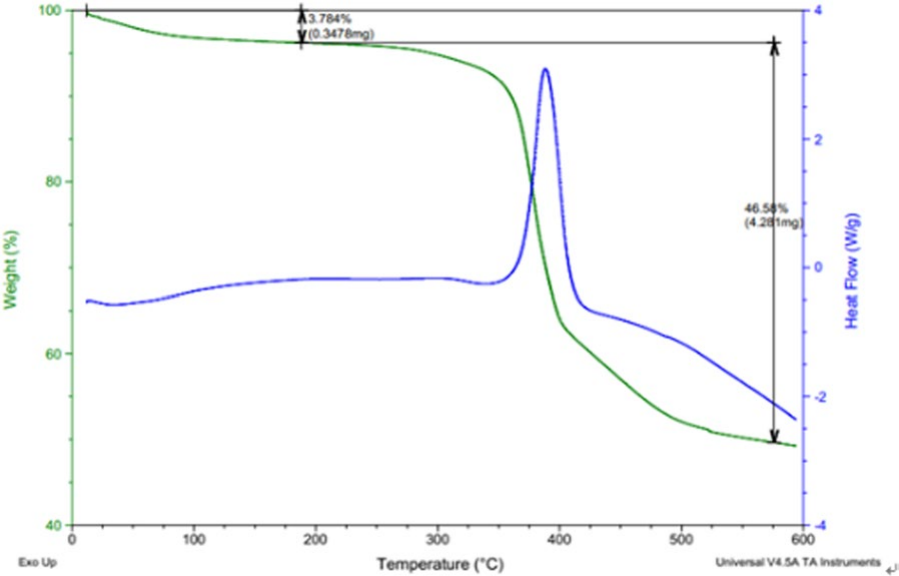
DSC-TG analysis results of Zn_2_(EBNB)_2_(BPY)_2_·2H_2_O (using NETZSCH STA 449 F1 Jupiter/10 °C/min)

The thermal gravimetric analysis of Zn_2_(EBNB)_2_(BPY)_2_·2H_2_O was carried out by DSC-TG in the temperature range of 0 °C to 600 °C. Figure 8 is the DSC-TG diagram of Zn_2_(EBNB)_2_(BPY)_2_·2H_2_O. It can be seen from the figure that Zn_2_(EBNB)_2_(BPY)_2_·2H_2_O can be stabilized to 350 °C. Zn_2_(EBNB)_2_(BPY)_2_·2H_2_O lost 3.78% of its first thermal weight in the temperature range of 50–200 °C, which can be attributed to the loss of guest molecules. However, in the temperature range of 350 °C ~ 560 °C, the structure collapses, resulting in 46.58% weight loss. The main component is ZnO.

## Study on sustained release of drug

### Determination of drug loading of Zn_2_(EBNB)_2_(BPY)_2_·2H_2_O

The maximum absorption peak of Methadone methanol solution is 292 nm.

Table 1 shows the relationship between the ratio of Methadone to carrier mass and the drug loading time to the drug loading of Zn_2_(EBNB)_2_(BPY)_2_·2H_2_O. It can be seen that with the increase of the ratio of Methadone to carrier mass (fixed carrier mass, increasing drug mass), the drug loading of Zn_2_(EBNB)_2_(BPY)_2_·2H_2_O increases. Also,the longer the action time, the higher the loading of Zn_2_(EBNB)_2_(BPY)_2_·2H_2_O. However, the highest loading of Zn_2_(EBNB)_2_(BPY)_2_·2H_2_O appeared on the 5th day under different drug to carrier mass ratio. This explains that the drug loading amount of Zn_2_(EBNB)_2_(BPY)_2_·2H_2_O reaches the maximum value at the 5th day of adsorption.

**Table 1:** Loading amount of Methadone (m_2_) on Zn_2_(EBNB)_2_(BPY)_2_·2H_2_O (m_1_) (n = 3)

M2/m1	Drug loading ratio m2/m1			
	1 day	3 day	5 day	7 day
1:1	0.175 ± 0.0014	0.188 ± 0.0020	0.221 ± 0.0025	0.201 ± 0.0021
3:1	0.177 ± 0.0019	0.201 ± 0.0007	0.217 ± 0.0019	0.209 ± 0.0013
5:1	0.182 ± 0.0022	0.233 ± 0.0012	0.256 ± 0.0010*	0.242 ± 0.0008
6:1	0.207 ± 0.0014	0.225 ± 0.0013	0.227 ± 0.0015	0.214 ± 0.0011

However, when the action time is extended to 7 days, the drug loading amount will decrease, which may be caused by the falling off of some drugs adsorbed on the surface of Zn_2_(EBNB)_2_(BPY)_2_·2H_2_O due to the long immersion time. This indicates that the best action time is 5 days. It can be seen from Table 1 that the best experimental condition of the mass ratio of the drug to the carrier is 5:1, the best action time is 5 days, and the highest drug loading can be obtained is 0.256 g/g carrier.

### In vitro release of Methadone loaded by Zn_2_(EBNB)_2_(BPY)_2_·2H_2_O

**Fig. 9:**
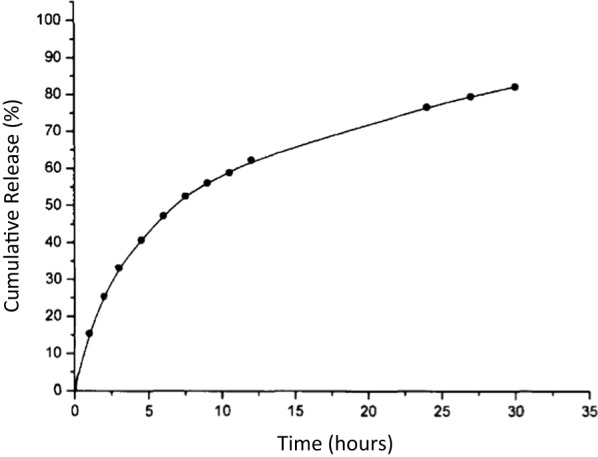
In vitro release curve of Methadone loaded by Zn_2_(EBNB)_2_(BPY)_2_·2H_2_O

As shown in Fig. 9, the drug release process is divided into two processes. The first 5 h of the drug release curve shows the characteristics of sudden release, and the sudden release of Methadone is 40.1% within 5 h. This is mainly due to the diffusion of the Methadone which adsorbed on the surface of Zn_2_(EBNB)_2_(BPY)_2_·2H_2_O into the medium. And then it enters a stable and slow release stage, those Methadone adsorbed in the Zn_2_(EBNB)_2_(BPY)_2_·2H_2_O channel is being released slowly. Within 30 h of stable release, the release amount of Methadone reached 79.85%, showing a significant slow-release effect.

### Cytotoxic test results of Zn_2_(EBNB)_2_(BPY)_2_·2H_2_O

In this study, normal growth cells (Zn_2_(EBNB)_2_(BPY)_2_·2H_2_O’s concentration of 0 μ g · ML^−1^) were used as the negative control group, and Zn_2_(EBNB)_2_(BPY)_2_·2H_2_O was used as the drug group to study the cytotoxicity of Zn_2_(EBNB)_2_(BPY)_2_·2H_2_O on HeLa cells in vitro. The results of in vitro cytotoxicity experiments shows that the survival rate of HeLa cells decreased with the increase of Zn_2_(EBNB)_2_(BPY)_2_·2H_2_O’s concentration after 36 h of exposure to different concentrations of Zn_2_(EBNB)_2_(BPY)_2_·2H_2_O. When the concentration of Zn_2_(EBNB)_2_(BPY)_2_·2H_2_O was less than 20 μ g·ml^−1^, the survival rate of HeLa cells was higher than that of the control group P > 0.05(Significant difference level). But when the concentration of Zn_2_(EBNB)_2_(BPY)_2_·2H_2_O is more than 20 μg·ml^−1^, the cell survival rate decreases with the increase of Zn_2_(EBNB)_2_(BPY)_2_·2H_2_O’s concentration. When the concentration of Zn_2_(EBNB)_2_(BPY)_2_·2H_2_O is 250 μg·ml^−1^, the cell survival rate reaches the lowest value.

## Conclusions

To sum up, Zn_2_(EBNB)_2_(BPY)_2_·2H_2_O can be synthesized by solvothermal method via (E)-bis(p-3-nitrobenzoic acid) ethylene ligand (C_16_H_10_N_2_O_8_). After drying and activation treatment, Zn_2_(EBNB)_2_(BPY)_2_·2H_2_O was loaded into Methadone with high drug loading. And the drug release curve shows that Zn_2_(EBNB)_2_(BPY)_2_·2H_2_O has slow-release function and can prolong Methadone’s work. In addition, Zn_2_(EBNB)_2_(BPY)_2_·2H_2_O has good biocompatibility and is expected to become an excellent drug carrier.

In this study, MOFs was introduced into the field of drug release. Some features of MOFs, such as high specific surface area; tailorable, designable, adjustable channel size and channel surface functionalization, are used to study the sustained-release mechanism of MOFs as a new drug delivery form. All of these provide reference information for the research and development of new dosage forms of anti-drug addiction.

## Experimental

### Chemistry

#### Sources of experimental materials

Methadone used in this experiment was purchased from the purchasing point designated by the Ministry of public security of China, the Academy of criminal Sciences of Shanghai Public Security Bureau. All the other experimental materials used are commercially available.

#### Synthesis of (E)–bis (p-3-nitrobenzoic acid) ethylene ligand (C_16_H_10_N_2_O_8_)

200 ml concentrated sulfuric acid was pouring into a 400 ml flask, and 100 ml fuming nitric acid was added under 0 °C ice water bath while stirring constantly. Then 10 g of p-chloromethylbenzoic acid was added in small parts. After 90 min of reaction, p-chloromethylbenzoic acid was completely dissolved into the mixed acid. All substances in the bottle was pouring into 600 ml of ice water, a large amount of white solid is precipitated immediately. The residual mix-acid was filtered and washed, and then the product was recrystallized in toluene solvent. The white crystal of 3-nitro-p-chloromethylbenzoic acid was obtained and dried in an oven (Yield: 89%). ^1^HNMR(200 MHz DMSO-*d*_*6*_)δ8.76(s,1H);8.36(d,1H);7.87(d,1H);5.03(s,2H).

50 ml anhydrous ethanol was poured into a 300 ml beaker, and 5.7 g KOH was dissolved in it. Then 5.00 g of 3-nitro-p-chloromethylbenzoic acid was poured into form brown precipitate, which is potassium salt of (E)–di (p-3-nitrobenzoic acid) ethylene. After reaction at room temperature for 45 min, the solid was vacuumed and dissolved in 70 ml water. After that, adding HCl to the aqueous solution to adjust pH = 1 then form a precipitate. The solid was collected and recrystallized with tetrahydrofuran solvent. The yellow crystal compound (E)-di(p-3-nitrobenzoic acid) vinyl (C_16_H_10_N_2_O_8_) was obtained and dried in an oven (Yield: 78%). ^1^HNMR(200 MHz DMSO-*d*_*6*_)δ7.62(s,2H);7.89(d,2H);8.52(d,2H);9.06(s,2H).

#### Synthesis of Zn_2_(EBNB)_2_(BPY)_2_·2H_2_O

The (E)-di(p-3-nitrobenzoic acid) ethylene ligand (C_16_H_10_N_2_O_8_) (0.15 mmol), 4,4′-bipyridine ligand (BPY,0.15 mmol), zinc nitrate (Zn(NO_3_)_2_·6H_2_O,0.15 mmol), ethanol 2 ml and H_2_O 10 ml were put into a 50 ml high-pressure reactor, and the pH was adjusted to 9, ultrasounded, 150 °C for 2 days. The rhombic yellow crystal (E)-di(p-3-nitrobenzoic acid) ethylene can be obtained after filtration and washing. (Yield: 60%) Element analysis: C 52.45, n 9.34, H 3.11, Zn 10.89%; theoretical value: C 52.37, N 9.40, H 3.02, Zn 10.98%. The product is composed of C_26_H_18_N_4_O_9_Zn (595.81).

#### Determination of structure

The suitable crystals of Zn_2_(EBNB)_2_(BPY)_2_·2H_2_O were selected for X-ray diffraction study. Diffraction data were collected on a Bruker SMART APEX-II CCD diffractometer equipped with a graphite-monochromated Mo-Kα radiation (λ = 0.71073 Å) by ρ-ω diffraction data at 298(2) K. All diffraction data through the SADABS software with multi-scan semi-empirical method of absorption correction. The structure were solved by direct methods and subsequent successive difference Fourier maps, and the structure was refined by full-matrix least-squares techniques on F^2^ using SHELXL-2014 program [22]. The crystal structure refinement software was Olex^2^(Version 1.2.7). The main crystallographic data are listed in the Tables 2, 3 and 4.

**Table 2:** Crystallographic data and structural correction conditions of 3-nitro-p-chloromethylbenzoic acid

Empirical formula	C_7_H_5_ClN_2_O_4_
Fomular weight	216.58
Crystalsystem	Monoclinic
Space group	P2(1)/c
Wavelength(Å)	0.71073
Temperature(K)	293(2)
a (Å)	7.5316(7)
b (Å)	17.0508(15)
c (Å)	14.1080(13)
α(^°^)	90.00
β(^°^)	100.6050(10)
γ(^°^)	90.00
V (Å^3^)	1780.8(3)
Z	8
Density (g/cm^3^)	1.515
μ(mm^−1^)	0.418
F(000)	880
θ range (°)	2.389 to 23.956
GOF on F^2^	1.036
R_1,_ wR_2_ [I > 2σ(I)]	R_1_ = 0.0661
	wR_2_ = 0.1719
R_1,_ wR_2_ (all data)	R_1_ = 0.1127
	wR_2_ = 0.2159

**Table 3:** Crystallographic data and structural correction conditions of (E)-di(p-3-nitrobenzoic acid) vinyl

Empirical formula	C16H13N2O9
Crystalsystem	Triclinic
Wavelength(Å)	0.71073
a(Å)	7.3757(4)
c(Å)	15.2903(10)
β(°)	82.9270(10)
V(Å3)	818.92(9)
Density (g/cm3)	1.545
F(000)	398
GOF on F2	1.029
Fomular weight	385.28
Space group	P-1
Temperature(K)	293(2)
b(Å)	7.7827(5)
α(°)	79.473(4)
γ(°)	72.069(4)
Z	2
μ(mm-1)	0.132
θ range (°)	2.717 to 28.347
R_1,_ wR_2_ [I > 2σ(I)]	R_1_ = 0.0444
	wR_2_ = 0.1161
R_1,_ wR_2_ (all data)	R_1_ = 0.0600
	wR_2_ = 0.1321

**Table 4:** Crystallographic data and structural correction conditions of Zn_2_(EBNB)_2_(BPY)_2_·2H_2_O

Empirical formula	C_26_H_18_N_4_O_9_Zn
Fomular weight	595.81
Crystal system	Triclinic
Space group	P-1
Wavelength(Å)	0.71073
Temperature(K)	293(2)
a(Å)	8.1862(9)
b(Å)	11.4220(14)
c(Å)	14.2863(17)
α(^°^)	96.8030(10)
β(^°^)	93.0450(10)
γ(^°^)	102.472(2)
V(Å^3^)	1290.8(3)
Z	2
Density (g/cm^3^)	1.533
μ(mm^−1^)	1.013
F(000)	608
θ range (°)	2.557 to 26.079
GOF on F^2^	1.008
R_1,_ wR_2_ [I > 2σ(I)]	R_1_ = 0.0499
	wR_2_ = 0.1067
R_1,_ wR_2_ (all data)	R_1_ = 0.0750
	wR_2_ = 0.1153

All hydrogen atoms were included in their calculated positions and treated as riding atoms with the U iso values assigned to 1.2U eq of their bonding carbon atoms and to 1.5U eq of their bonding oxygen atoms, respectively.

CCDC: 1545065 for 3-nitro-p-chloromethylbenzoic acid.

CCDC: 912099 for (E)-di(p-3-nitrobenzoic acid) vinyl.

CCDC: 910300 for Zn_2_(EBNB)_2_(BPY)_2_·2H_2_O.

#### Methadone loaded into Zn_2_(EBNB)_2_(BPY)_2_·2H_2_O

Accurately weigh proper amount of Methadone, and prepar the solution with methanol solvent. The maximum absorption peak is 292 nm. Then the concentration gradient of the standard solution was prepared, and the standard curves of absorbance (A) and concentration (c) at this wavelength were established. The standard curve is suitable for drug loading environment.

In the same way as the above operation, the appropriate concentration of Methadone solution was prepared with phosphate buffer (PBS, pH = 7.4) as the solvent, and the standard curves of absorbance (A) and concentration (c) were established with PBS as the blank control. The standard curve is suitable for drug releasing environment.

Accurately weigh 2 mg of dried and activated Zn_2_(EBNB)_2_(BPY)_2_·2H_2_O, add in 1 ml of methanol solution which contain 10 mg of Methadone, mixing under ultrasound, acting for 24 h. Then centrifugate the reaction solution (10000 rpm, 10 min), take 100 μl of the supernatant and dilute it to 10 ml (100 times), measure its absorbance at 292 nm, then calculate the drug loading of Zn_2_(EBNB)_2_(BPY)_2_·2H_2_O.

$$\text{Drug}{\kern 1pt} \,\text{loading}\,\text{ = }\,\left( {\text{Amount}\,\text{of}\,\text{drug}\,\,\text{in}\,\,\text{MOF/Total}\,\,\text{mass}\,\,\text{of}\,\,\text{MOF}\,} \right)\,{ \times }{\kern 1pt} {100\% }$$

#### In vitro release of methadone from Zn_2_(EBNB)_2_(BPY)_2_·2H_2_O

Take 2 mg of Zn_2_(EBNB)_2_(BPY)_2_·2H_2_O loaded with Methadone and put it into 20 ml PBS buffer solution, which is the experimental group. Take another 1.16 mg of Zn_2_(EBNB)_2_(BPY)_2_·2H_2_O and put it into 20 ml PBS buffer, which is the blank group. Under (37 ± 1 °C) constant temperature oscillator (oscillation speed: 100r·Min^−1^), take 1 ml of solution every 12 h and add in equal amount of fresh PBS buffer, then centrifugate the solution (12000 rpm, 20 min), after that take the supernatant to determine its absorbance by ultraviolet spectrum. The content of Methadone in the solution was calculated according to the established Methadone standard curve, and the relationship curve between cumulative release and time was drawn. The in vitro releasing performance of Methadone from Zn_2_(EBNB)_2_(BPY)_2_·2H_2_O was investigated accordingly.

### Cytotoxicity of Zn_2_(EBNB)_2_(BPY)_2_·2H_2_O

HeLa cells were cultured in RPMI 1640 medium, which contains 10% fetal bovine serum. HeLa cells were used only at logarithmic growth stage and good growth state. Cells were digested with 0.25% trypsin and centrifuged to precipitate. RPMI 1640 culture medium which contains 10% fetal bovine serum was used to prepare cell suspension with a cell density of 1 × 10^5^ cells·ml^−1^. Then it was inoculated into 96 well culture plate (104 cells per well, six multiple holes, each hole was 100 μ L.).

Then the culture plate was transferred to the incubator, and 100μ L RPMI 1640 culture medium was replaced at 37 °C, 5% CO_2_ and saturated humidity for 24 h. The experiment was divided into three groups: blank control (without cells), negative control (without cells, without Zn_2_(EBNB)_2_(BPY)_2_·2H_2_O), Zn_2_(EBNB)_2_(BPY)_2_·2H_2_O (with cells, with Zn_2_(EBNB)_2_(BPY)_2_·2H_2_O). Then, 100μL solution of each group was added into make the mass concentration of 200, 100, 50, 25, 12, 6 μg·ml^−1^ respectively, then continue to culture for 48 h. Then change 100μL RPMI 1640 culture solution for each hole and 20μL MTT solution (5 mg·ML^−1^) was added. Shake it on a micro oscillator for 20 min, continue to culture for 24 h. Remove the culture solution, added in 150μL DMSO for each hole, absorbance (A) at 490 nm was determined by enzyme-linked immunosorbent assay. The cell survival rate can be calculated by accordingly.

$$Survival\,rate\% \, = \,\left( {Drug\,group\,\text{ - }\,Blank\,group} \right)\,\text{/}\,\left( {Negative\,control\,group\,\text{ - }\,Blank\,group} \right)\, \times \,100\% .$$

## Data Availability

The datasets used and/or analysed during the current study available from the corresponding author on reasonable request.

## Abbreviations

MOFs: Metal organic Frameworks
EBNB: (E)-di(p-3-nitrobenzoic acid) vinyl
BPY: Bipyridine
*SEM*: Scanning electron microscope
XRD: Diffraction of x-rays
DSC-TG: Differential scanning calorimetry-Thermogravimetric analysis
^1^HNMR: Proton nuclear magnetic resonance
CCDC: The Cambridge Crystallographic Data Centre
PBS: Phosphate buffer saline
RPMI: Culture medium of Roswell Park Memorial Institute
MTT: Thiazolyl Blue Tetrazolium Bromide
